# Topical Delivery of Vitamin D3: A Randomized Controlled Pilot Study

**Published:** 2014-03

**Authors:** Mir Sadat-Ali, Dalal A. Bubshait, Haifa A. Al-Turki, Dakheel A. Al-Dakheel, Wissam S. Al-Olayani

**Affiliations:** College of Medicine, University of Dammam, King Fahd Hospital of the University, AlKhobar, Saudi Arabia

**Keywords:** Vitamin D, route of administration, deficiency, Medication Burden

## Abstract

**Background and Objective:**

The purpose of the present study is to explore the assessment if the transdermal delivery of vitamin D is feasible.

**Methods:**

In 50 female Medical students, this study was conducted. Age, weight and height was taken, a detailed history and clinical examination was performed. Blood was drawn for 25 Hydroxy Vitamin D3 (25OHD) level. Two women had >30 ng/mL of 25OHD and was excluded from the study. The participants were divided into two groups of 24 in each arm. All participants equivocally agreed not to change their dietary habits and life style till the study was over. The study group of women were asked to apply; Top-D (Aloe Vera based- Vitamin D3) (Patency Pending) was developed at King Fahd Hospital of the University, AlKhobar with each gram of the Top-D cream delivering 5000 IU of vitamin D3. The second group used 1 gram of Aloe vera gel. The participants had no knowledge to which group they belong. A second blood sample was taken at the end of 3 months and the data was analyzed.

**Results:**

The data of 48 women was available for analysis. The average age was 22.58 ± 1.95 years. The mean pre-treatment 25OHD in the study group was 12.05 ng/Ml ± 6.54 and post-treatment was 37.95 ng/mL ± 6.43 (P=0.001, CI<28.582 ). In control group pre-treatment 25OHD was 11.4 ng/mL ± 3.97 and post-treatment was 10.58ng/mL ± 3.03.

**Conclusions:**

This randomized control study shows that vitamin D3 can safely be delivered through the dermal route. This route could be exploited in treating vitamin D deficiency.

## INTRODUCTION

Vitamin D (VD) is a fat-soluble essential vitamin that is required on a daily basis to treat, either rickets in children or osteomalacia in the adults. The first scientific description of a vitamin D-deficiency, namely rickets, was provided in the 17th century by both Dr. Daniel Whistler (1) and Professor Francis Glisson (2), but in 1822, Sniadecki (3) was the first to recognize and report the association of rickets with a lack of sunlight exposure. It had to wait for another 80 years before the work of Mellanby and McCollum led to the discovery of vitamin D in 1921 (4). Years have passed and still we are investigating the functions and benefits of VD.

There is confusion regarding the terminology of VD and its dosage. There are two principle types of VD, Vitamin D2 and D3 and other active analogs. Ergocalciferol (D2) is derived from sources such as fortified milk, herring, mackerel, tuna, salmon, sardines, eggs, fortified cereals and baked goods, while Vitamin D3, otherwise known as cholecalciferol, is a pro-hormone and essential nutrient produced in the skin with exposure to UV rays, animal products and fortified foods. Vitamin D3 can be produced photochemically by the action of sunlight or ultraviolet light from the precursor sterol 7-dehydrocholesterol which is present in the epidermis of the skin. It can also be consumed in the form of fish oil, or eaten in foods such as eggs or fish. An analog of VD is produced synthetically.

The deficiency of VD occurs due to inadequate exposure to the sun or due to its low content in the diet. As early as 1980’s it was found that ethnic Saudi population has low vitamin D. Extensive work of Sedrani *et al*. (5, 6) has shown that deficiency exists not only in the winter but the summer months due to non-exposure to the sun. Al-Turki *et al*. (7), Sadat-Ali *et al*. (8) found in the healthy Saudi population the VD deficiency in about 40-60% of men and women in over ≥50 years. Recent studies put the deficiency of vitamin D to be 95-100% (9, 10). Deficiency of vitamin D is noticed also in women who is prescribed the correct doses of vitamin D due to non compliance and also due to medication burden (11-13).

With the objective to assess the transdermal delivery of VD using aromatic oils and aloe vera gel as permeation enhancers, this prospective RCT was conducted.

## METHODS

After obtaining the approval from the research and ethical committee of University of Dammam and informed consent from 50 healthy unmarried, female students the study was commenced. Participants picking up sealed envelope did randomization.

Age, weight and height was taken, a detailed history, meticulous clinical examination was performed to rule out any diseases and as a standard hospital protocol complete blood picture, serum calcium, phosphorous, alkaline phosphatase, Parathormone and 25 Hydroxy Vitamin D3 (25OHD). Two women had vitamin D3 level of >30 ng/mL of 25OHD and was excluded from the study. 25 Hydroxy Vitamin D3 was measured in house by chemiluminescence immunoassay (CLIA) and ≥30 ng/mL was taken as normal, 21-29 ng/mL as insufficiency and ≤20 ng/mL as deficiency. The participants were divided into two groups of 24 in each arm. All participants equivocally agreed not to change their dietary habits and life style till the study was over. The study groups of women were asked to apply; Top-D (Aloe Vera based- Vitamin D3) was developed at King Fahd Hospital of the University, AlKhobar with each gram of the Top-D cream delivering 5000 IU of vitamin D3. The second group used 1 gram of Aloe vera gel. The participants had no knowledge to which group they belong. A second blood sample was taken at the end of 3 months and the data was entered in the database and analyzed using a *t*-test to compare means between the two groups, for all the parameters tested before and after topical use of VD and AVG. All tests were performed using SPSS (Statistical Package for the Social Sciences), version 14.0, Chicago, Illinois.

A *p* value of <0.05 was considered statistical significant with Confidence Interval (CI) of 95%.

## RESULTS

**Figure 1:**
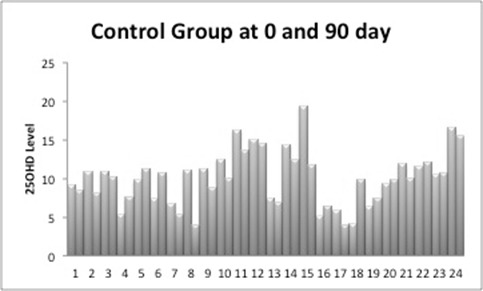
25OHD in Control Group at pre and post placebo treatment.

**Figure 2:**
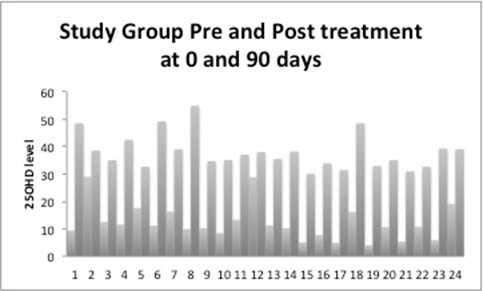
25OHD levels pre and post treatment with Topical Vitamin D.

The data of 48 women was available for analysis. The mean age was 22.58 ± 1.95 with Body Mass Index (BMI) of mean 19.95 ± 3.15 kg/M^2^. Table 1 gives the data of age, BMI and blood levels. In the study group the average BMI was 19.91 ± 2.93 kg/M^2^ and control group was 20.0 ± 3.41 kg/M^2^ (*P* value <0.2). The average 25OHD in the study group pre-treatment was 12.05 ng/mL ± 6.54 and post-treatment was 37.95ng/mL ± 6.43 (*P*≤0.0001). In the control group the pre-treatment 25OHD was 10.4 ng/mL ± 3.97 and post-treatment was 9.58 ng/mL ± 3.03. Figure 1 gives the comparison between the pre and post treatment of control group of participants who was given placebo while Figure 2 shows that of the study group. In the control group there was no statistically change between the groups where as in the study group women who had lower level of 25OHD showed marked improvement in the levels of level of 25OHD. The comparison between the two groups is given in Table 2.

**Table 1:** Age, BMI and Blood Levels of all Participants

Parameter	Average	Range

Age (Years)	22.58 ± 1.95	(19-27)
BMI Kg/M^2^	19.95 ± 3.15	13.8-27.2
Hemoglobin level g/L (11.6-14g/L)	11.97 ± 0.97	9.4-13.3
Calcium mg/dL (8.5 -10.2 mg/dL)	9.97 ± 0.54	8.2-10.5
Phosphorus mg/dl (2.4 to 4.0 mg/dL)	3.74 ± 0.54	2.9-4.8
Alkaline Phosphatase U/L (40-140IU)	78.37 ± 26.61	70-135
Parathormone pc/L (1.3-6.8 pc/L)	8.24 ± 3.9	2.74-17
25OHD ng/mL (≥30ng/mL)	11.22 ± 5.41	4-28.9

**Table 2:** Blood Levels of Both groups Pre and Post treatment

	Study Group	Control Group	*P* Value

Hemoglobin level g/L	11.92 ± 0.87	11.26 ± 0.92	0.2
Calcium mg/dl	9.0 ± 0.6	8.95 ± 0.48	0.6
Phosphorus mg/dl	3.78 ± 0.66	3.7 ± 0.61	0.7
Alkaline Phosphatase U/L	74.91 ± 24.84	82.25 ± 28.19	0.01 CI < -7.148
Parathormone	8.33 ± 4.13	9.15 ± 3.74	0.2
25 OHD pretreatment ng/mL	12.05 ± 6.54	11.4 ± 3.97	0.4
25OHD Post-treatment ng/mL	37.95 ± 6.43	10.58 ± 3.03	0.001 CI <28.5828

## DISCUSSION

Our study shows that our formulation of vitamin D3 can safely and effectively be delivered by dermal route reducing the incidence of non-compliance of oral route. Most common routes of administration of VD is either oral or recently invasive injectable route. For any drug large proportions of oral prescriptions are never taken at all (14). Recent estimates for noncompliance range from study to study with ranges of 62 to 84 percent using electronic monitoring (15, 16) hence we believe that in the young and elderly oral route can be by passed by the use of transdermal route.

The use of skin to deliver oils and balms is known to mankind for many centuries, but physicians used creams and lotions to treat only skin diseases. Application of topical products to the skin can act locally or pass into the systemic circulation, or do both.

Since its first approval by the US FDA scopolamine for motion sickness in December 1979 (17), many other were developed for transdermal and topical drug delivery (18-23).

Although the stratum corneum is an efficient barrier, some chemical substances are able to penetrate it and to reach the underlying tissues and blood vessels. These successful substances have to be lipophilic and VD is a fat-soluble which should be able to cross the skin barrier. Efforts are up to investigate and modify the structure at the cellular level of the stratum corneum in maximizing the absorption. Morrow *et al*. (24) in a review of literature described five different methods by which drug delivery could be achieved. The most ideal penetration enhancer discovered to date is undoubtedly water. Hydration of the stratum corneum has been shown to increase the penetration of both hydrophilic and hydrophobic drugs (25, 26). In this study we used a composition of aromatic oils and glycerine as permeation enhancer, which worked without complications.

At present VD supplementation is available in the oral and injection forms and both have their own limitations. The compliance of oral VD and calcium supplementation is reported between 20-60% (27-30). We believe that supplementation by topical route may increase the compliance among patients and our study further shows that with the daily dosage within a 90-day period the 25OHD returned to a minimum normal level of 30 ng/mL.

The limitation of our study is that the numbers are smaller in both the groups keeping in regard to the deficiency of vitamin D. We believe that we could have increased the number of patients. In conclusion the results of our study dictate us to state that topical route of vitamin D is possible, efficacious and safe.
